# Nanomaterials for diabetic wound healing: Visualization and bibliometric analysis from 2011 to 2021

**DOI:** 10.3389/fendo.2023.1124027

**Published:** 2023-01-25

**Authors:** Jun Zhang, Hongyan Liu, Tingting Che, Yin Zheng, Xixi Nan, Zhongming Wu

**Affiliations:** ^1^ NHC Key Laboratory of Hormones and Development, Tianjin Key Laboratory of Metabolic Diseases, Chu Hsien-I Memorial Hospital & Tianjin Institute of Endocrinology, Tianjin Medical University, Tianjin, China; ^2^ Department of Endocrinology, Shandong Provincial Hospital Affiliated to Shandong First Medical University, Jinan, China

**Keywords:** diabetic non-healing wound, knowledge map, research trends, web of science, bibliometric

## Abstract

**Background:**

Nanomaterials have recently been shown to have a considerable advantage in promoting wound healing in diabetic patients or animal models. However, no bibliometric analysis has been conducted to evaluate global scientific production. Herein, this study aimed to summarize the current characteristics, explore research trends, and clarify the direction of nanomaterials and diabetic wound healing in the future.

**Methods:**

Relevant publications from 2011 to 2021 were collected from the Web of Science Core Collection on October 3, 2022. VOSviewer, CiteSpace, bibliometrix-R package, Origin 2021, and Microsoft Excel 2019 were used for bibliometric and visualization analyses.

**Results:**

We identified 409 publications relating to nanomaterials and diabetic wound healing. The number of annual productions remarkably increased from 2011 to 2021, with China and Shanghai Jiao Tong University being the most productive. The most prolific authors were Hasan Anwarul. The leading journal was the International Journal of Biological Macromolecules, with 22 publications. The most popular keywords were “nanoparticles,” “delivery,” “*in vitro*,” “electrospinning,” “angiogenesis,” and “antibacterial.” Keyword burst analysis showed “cerium oxide,” “matrix metalloproteinase 9,” “composite nanofiber,” “hif 1 alpha,” and “oxide nanoparticle” were emerging research hotspots.

**Conclusion:**

We found there has been a great progress in the application of nanomaterials in diabetic wound healing from 2011 to 2021. Although many researchers and institutions from different countries or regions contributed contributed to publications, it will be helpful or the development of this field if the degree of international cooperation can be enhanced. In the future, nanomaterials with powerful antioxidant and antibacterial qualities and promoting angiogenesis are the research hotspots.

## 1 Introduction

Diabetes mellitus (DM) is the most prevalent chronic metabolic disease characterized by hyperglycemia due to insulin deficiency or insulin resistance (1). The 10th edition IDF Diabetes Atlas estimated that approximately 537 million individuals live with diabetes worldwide (2). China has the highest number of patients with diabetes, which may reach over 174 million by 2045 (3). Besides sustained hyperglycemia, various complications occur, including diabetic ulcers, diabetic cardiomyopathy, and diabetic nephropathy. Diabetic foot ulcers (DFU) or diabetic non-healing wounds are catastrophic complications, which put a great social and economic burden on patients and the healthcare system (4). DFU affects 19%–34% of patients with DM, leading to 15%–25% of amputations and increasing the five-year death rate to 80% (5–7). A seven-year single-center retrospective review reported that the average total cost per patient was ¥21,826.91 ($3089.14) (8).

Normal wound healing consists of four successive and overlapping stages: hemostasis, inflammation, proliferation, and remodeling, which involve many types of cells and cytokines (9). However, diabetic wound healing usually does not progress due to the complex microenvironment with hyperglycemia, hypoxia, poor angiogenesis, and bacterial infection (10). Conventional treatment, including debridement, dressing, wound off-loading, infection control, vascular assessment, and glycemic control were ineffective, with 14%–20% of patients eventually undergoing low limb amputations (11, 12). Development of new treatments, such as nanotechnology, are urgently needed to improve patients’ quality of life. Recently, many studies have been performed to verify that nanomaterials with different biological characteristics can get encouraging results in diabetic wound healing (13–15). Some nanomaterial-based wound dressings, such as hydrogels and nanofibers, provide a wet atmosphere by imitating the extracellular matrix for wound healing and enable the cell migration and proliferation by loading some drugs, protein and cytockines; Some nanomaterials, such as bioglass nanoparticles and metal nanoparticles, are of great angiogenic and antibacterial activities to improve the antibacterial effect and reduce the abuse of antibiotics by acting as the smart delivery system (16–18). Currently, several reviews have discussed the advances in the link between nanomaterials and diabetic wound healing, but comprehensive evaluation of the research status in this field is challenging.

Bibliometrics is an interdisciplinary subject that can be applied to conduct quantitative and qualitative analyses of publications by using mathematical and statistical methods (19). It includes the contributions and influence of different authors, countries/regions, institutions, journals, and trends (20). To the best of our knowledge, no bibliometric analysis has summarized current research hotspots. This study aimed to examine publication trends in nanomaterials and diabetic wound healing from 2011 to 2021 and provide future research direction for new researchers *via* the Web of Science Core Collection (WoSCC).

## 2 Materials and methods

### 2.1 Data collection and retrieval strategies

**Figure 1:**
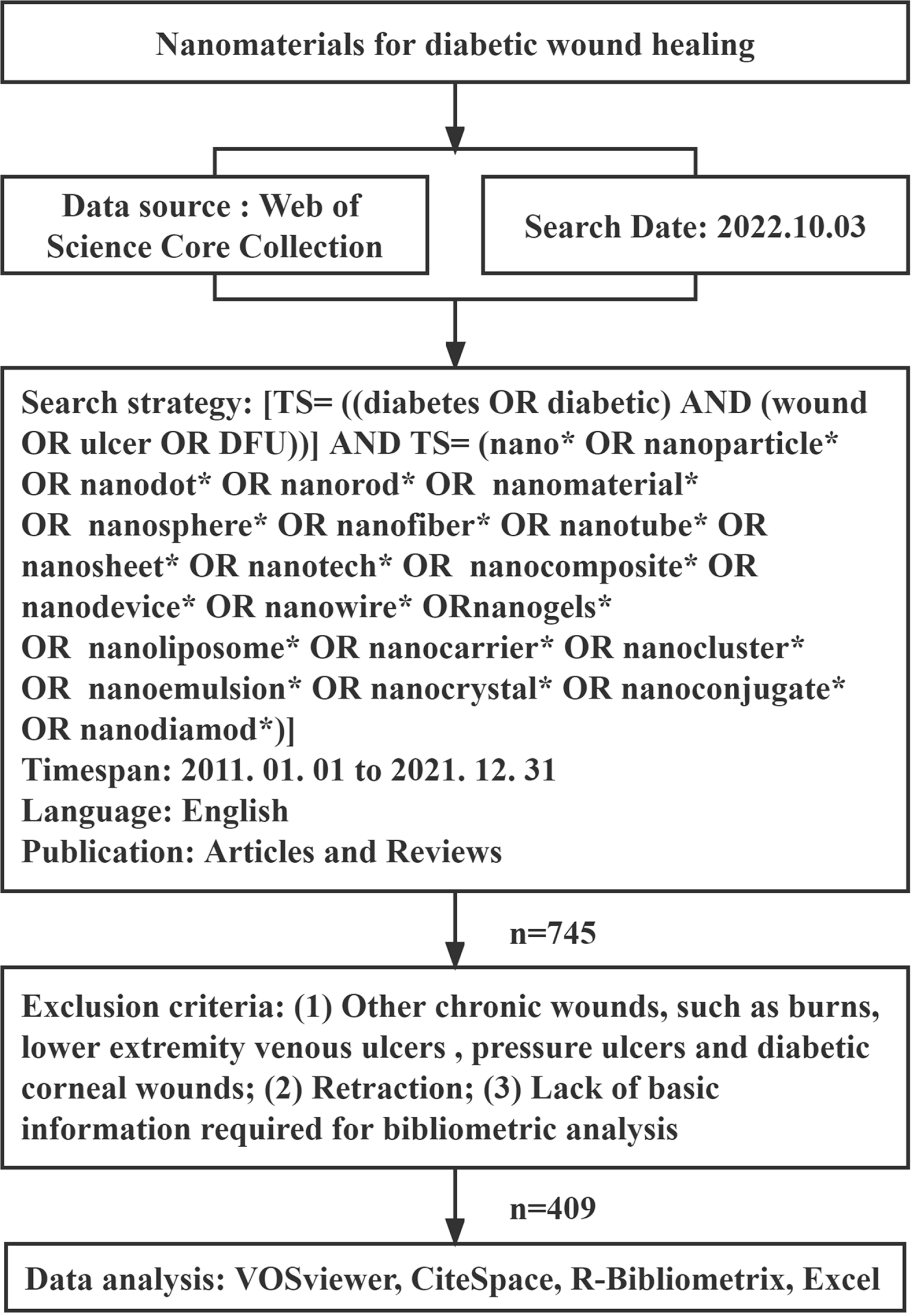
Flowchart for including and excluding publications.

We collected publication data and downloaded them as plain text from WoSCC on October 3, 2022. Data included the article title, authors, journal title, publication year, institutions, keywords, citation frequency, and other basic information. **Figure 1** shows data acquisition and retrieval strategies.

### 2.2 Inclusion and exclusion criteria

The accuracy of data must be ensured for analysis. Two researchers independently retrieved literature, and disagreements were resolved by discussing with outside parties, if necessary.

#### 2.2.1 Inclusion criteria

(1) The research topic should be the application of nanomaterials in diabetic ulcers or diabetic wounds. (2) The document type is an article or a review article. (3) The document language is limited to English. (4) The study was published from January 1, 2011, to December 31, 2021.

#### 2.2.2 Exclusion criteria

(1) Irrelevant documents, (2) other chronic wounds, such as burns, lower extremity venous ulcers, pressure ulcers, and diabetic corneal wounds, (3) retraction, (4) lack of basic information required for bibliometric analysis.

### 2.3 Data analysis and visualization

CiteSpace is Java-based bibliometric software, a widely used tool for the visual exploration of scientific literature, and helps discover research hotspots and future trends (21). We used CiteSpace (6.1.4) to perform dual-map overlay of journals, produce clustering analysis of keywords and co-cited references by using the log-likelihood ratio (LLR) algorithm, create a timeline view of keywords and co-cited references, and conduct keywords and co-cited references with the strongest burst.

In 2009, Van Eck and Waltman of The Centre for Science and Technology Studies at Leiden University in the Netherlands developed VOSviewer, a Java-based free software (22). VOSviewer (1.6.18) was used to visualize the co-citation network of countries, institutions, and authors. In the visual map, the node size indicated numbers or frequency, and the line thickness represented the strength of the link.

The bibliometrix R package (https://www.bibliometrix.org) was used to generate the distribution map of global publications. Microsoft Excel 2019 and Origin 2021 were used to display annual publications produced, international links of countries, and changes in yearly volume in China, India, and the USA.

## 3 Results

### 3.1 General information

**Figure 2:**
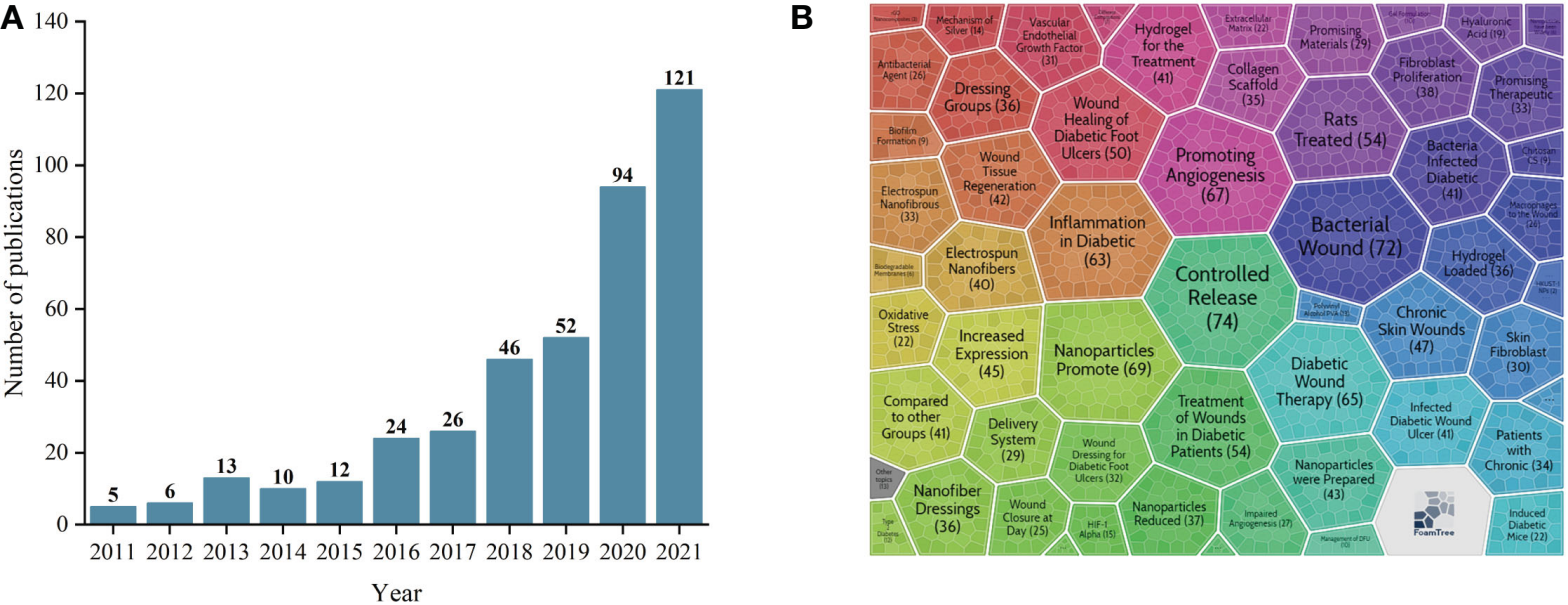
Annual number of publications **(A)** and theme clusters for the nanomaterials in diabetic non-healing wound by Carrot2 **(B)**.

We included 409 documents meeting the inclusion and exclusion criteria from the Web of Science database in our bibliometric analysis. **Figure 2A** shows the annual production of publications. Only a few articles were published in this field. Despite only five papers published in 2011, the number increased to 121 in 2021, with an increase of 2330%. Two phases were observed in the growth of literature published in the last decade: slow growth from 2011 to 2015, followed by rapid growth from 2016 to 2021. Carrot2 is an application for text clustering algorithms and organizing search results into topics. We divided 409 documents into 57 theme clusters for the Carrot2 analysis. We found that the top topics were “controlled release,” “bacterial wound,” “promoting angiogenesis,” “nanoparticles promote,” and “diabetic wound therapy” (**Figure 2B**).

### 3.2 Contributions of countries/regions

**Figure 3:**
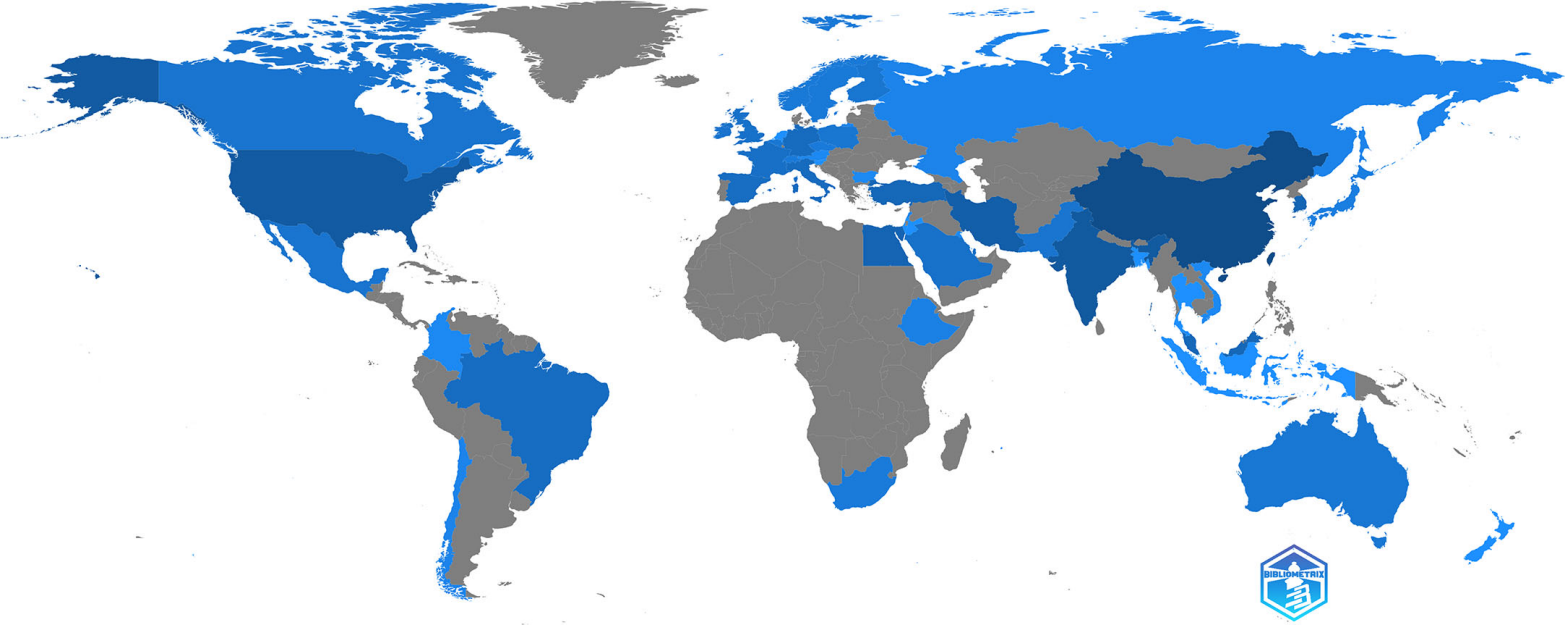
Map of global publications.

**Figure 4:**
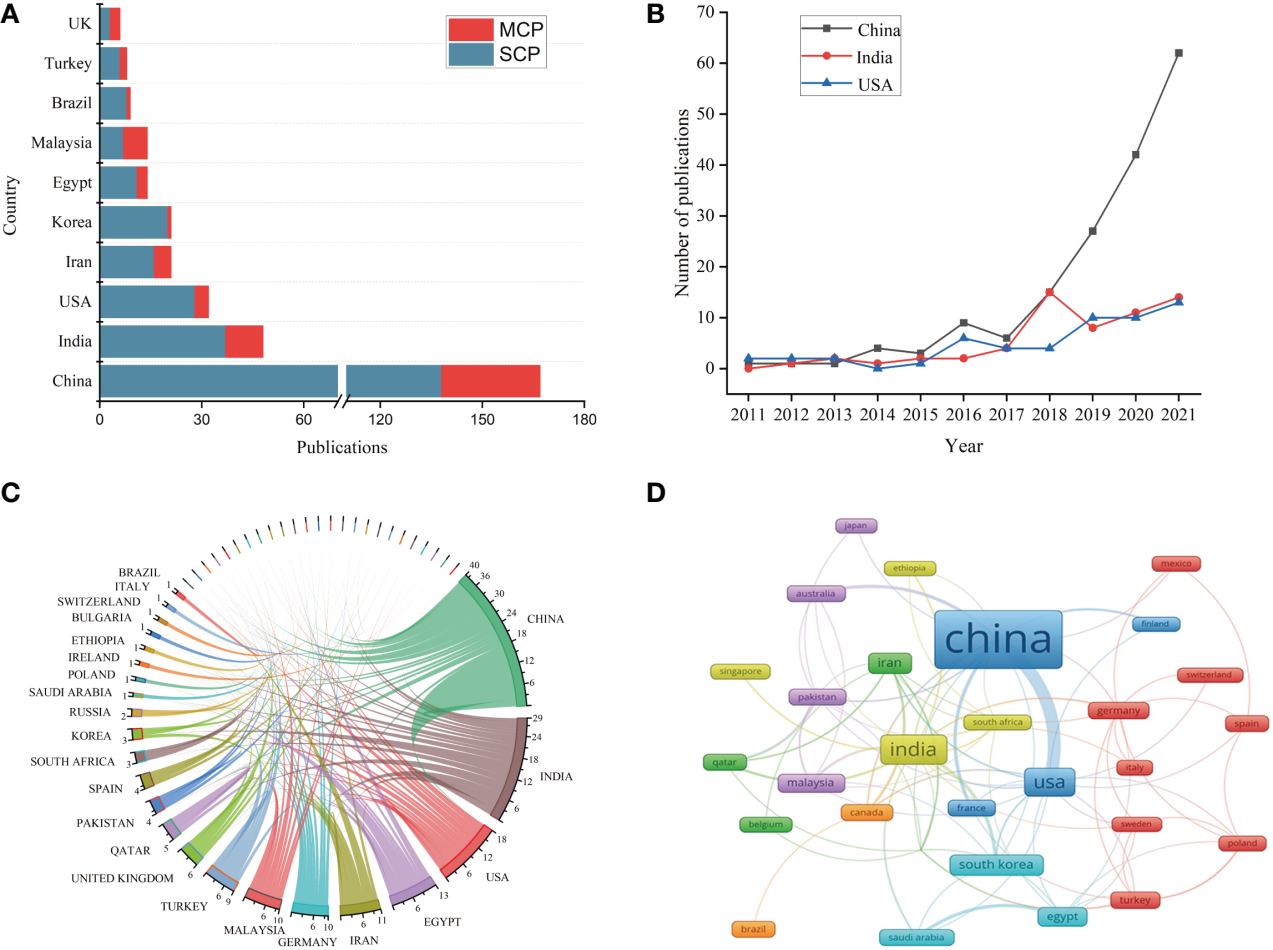
The distribution of single or multiple country papers in top 10 countries **(A)**; annual number of publications in China, India and USA **(B)**; The international cooperation networks between countries/regions **(C)**; VOSviewer network map of countries/regions **(D)**.

Fifty-one countries or regions contributed to publications in nanomaterials and diabetic wound healing. The distribution of published documents was shown on a world map, with the blue intensity representing the total number of publications (**Figure 3**). **Figure 4A** and **Table 1** show that China published the most number of papers (n = 167, 40.83%), followed by India (n = 48, 11.74%), the USA (n = 32, 7.82%), Iran (n = 21, 5.13%), South Korea (n = 21, 5.13%), Egypt (n = 14, 3.42%), and Malaysia (n = 14, 4.42%). Other countries or regions published no more than five papers. In 2018, China’s annual publication volume was significantly higher than that of India and the USA (**Figure 4B**). Among the top 10 countries, China also had the highest number of single-country papers, whereas the ratio of multiple-country papers ranked behind the USA and Brazil (**Table 1**). Moreover, the number of publications in China was significantly higher than that in other countries.

**Table 1:** Total number of publications, single country publications, and multiple country publications of top 20 Countries by corresponding authors.

Country	Articles	Percentage of 409 articles	SCP	MCP	MCP Ratio
China	167	40.83%	138	29	0.174
India	48	11.74%	37	11	0.229
USA	32	7.82%	28	4	0.125
Iran	21	5.13%	16	5	0.238
Korea	21	5.13%	20	1	0.048
Egypt	14	3.42%	11	3	0.214
Malaysia	14	3.42%	7	7	0.5
Brazil	9	2.2%	8	1	0.111
Turkey	8	1.96%	6	2	0.25
United Kingdom	6	1.45%	3	3	0.5

**Figures 4C, D** shows countries or regions with co-occurrence network. The size of the nodes represented the number of papers, and the line thickness showed cooperation strength. Of 51 countries, China and the USA had the strongest links. Accordingly, China was the most influential in the field based on publications and centrality.

### 3.3 Contributions of institutions

In the field of nanomaterials and diabetic wound healing, 699 institutions published 409 articles. **Table 2** shows the top ten contributing institutions. Shanghai Jiao Tong University published the most papers (n = 17), followed by the Chinese Academy of Sciences (n = 16), Shanghai Normal University (n = 10), Sichuan University (n = 10), and Tehran University of Medical Sciences (n = 10). Other institutions published fewer than ten articles. Of the top ten institutions, eight were from China, and the remaining were from Iran and Qatar.

**Table 2:** The top 10 institutions in the field of nanomaterials and diabetic wound healing.

Institution	Articles	Total citations	Country
Shanghai Jiao Tong Univ	17	551	China
Chinese Acad Sci	16	640	China
Shanghai Normal Univ	10	388	China
Sichuan Univ	10	261	China
Univ Tehran Med Sci	10	466	Iran
Jinzhou Med Univ	9	166	China
Qatar Univ	8	569	Qatar
Zhejiang Univ	8	443	China
Nankai Univ	7	218	China
Chang Gung Univ	7	296	China

**Figure 5:**
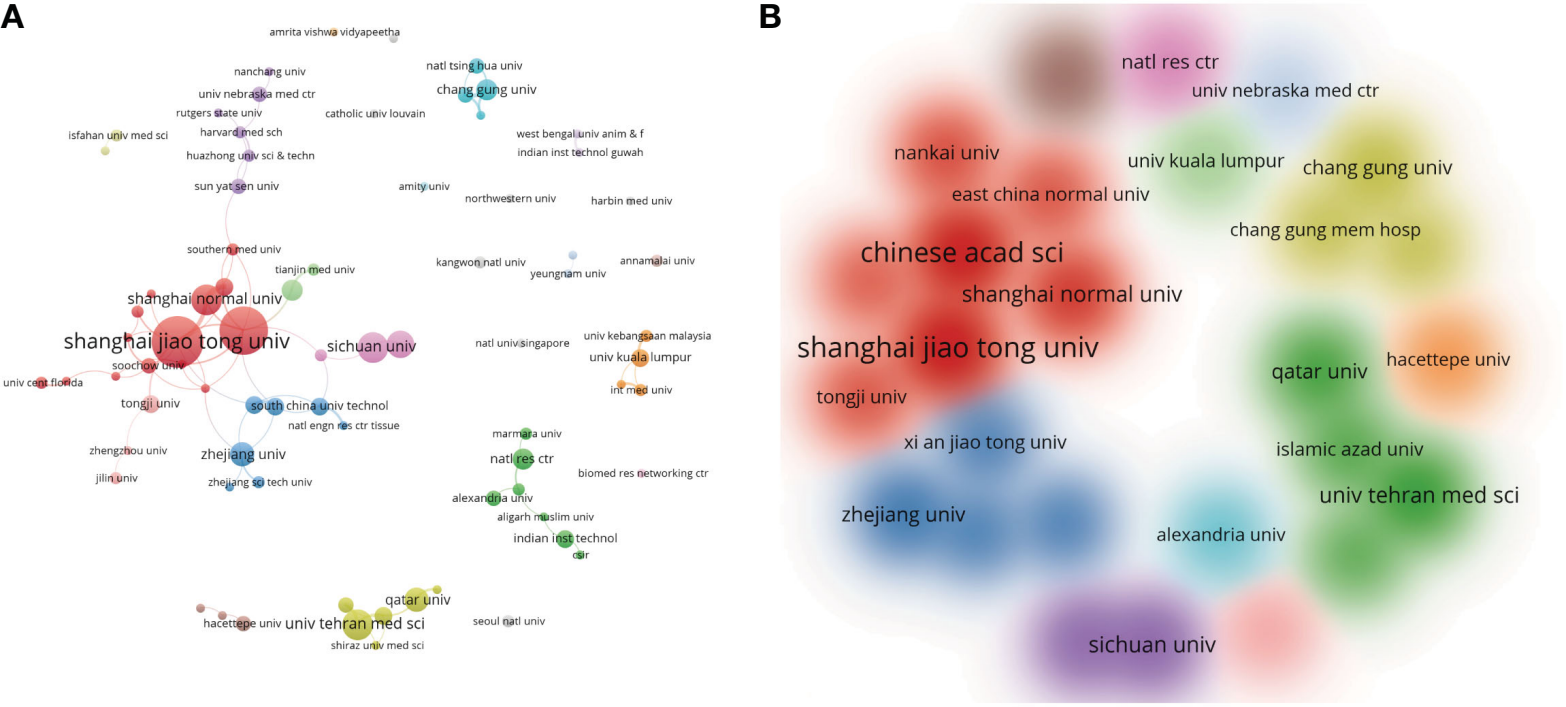
VOSviewer network map of institutions **(A)**; Density distribution map of institutions **(B)**. Minimum number of articles published by the institution was set to 5.

We used VOSviewer to demonstrate the co-authorship network of institutions (**Figure 5**). The minimum number of articles published by the institution was set to 5, and 13 clusters consisting of 27 institutions met the thresholds. Some of the 27 institutions were connected with others, with the largest set of connected clusters consisting of 11 institutions, of which Shanghai Jiao Tong University, Chinese Academy of Science, and Shanghai Normal University were the core. Research institutions in China were the leading organizations in nanomaterials and diabetic wound healing.

### 3.4 Authors

We retrieved 409 articles with 2508 authors, with an average of 6 authors per article. **Table 3** lists 13 of the most effective authors. Xu He and Hasan Anwarul were the most prolific scholars, with 8 papers each, followed by Ke Qinfei (n = 7). In terms of total citations, Hasan Anwarul ranked first with 569 citations, followed by Jayakumar (n = 402) and Augustine Robin (n = 401). Although Xu He and Hasan Anwarul had the same number of publications, the average citations per paper of Hasan Anwarul (71.13 times) were significantly higher than those of Xu He (40.88 times).

**Table 3:** The top 13 authors in the field of nanomaterials and diabetic wound healing.

Rank	Author	Papers	Total citations	Average citations per paper	H-index	G-index	Ms-index
1	Hasan Anwarul	8	569	71.13	7	8	1.4
2	Xu He	8	327	40.88	7	8	1.167
3	Ke Qinfei	7	309	44.14	7	8	1
4	Chang Shang-hung	6	208	34.67	6	6	0.667
5	Hung Kuo-chun	6	208	34.67	6	6	0.667
6	Juang Jyuhn-huang	6	208	34.67	6	6	0.667
7	Liu Shih-jung	6	208	34.67	7	6	0.667
8	Yi Zhengfang	5	272	54.40	5	5	0.883
9	Augustine Robin	5	401	80.20	5	5	1
10	Chen zhenhua	5	108	21.60	4	5	1.333
11	Mei Xifan	5	108	21.60	4	5	1.333
12	Jayakumar R	5	402	80.40	5	5	0.455
13	Zhang Xinge	5	265	53.00	4	4	0.444

**Figure 6:**
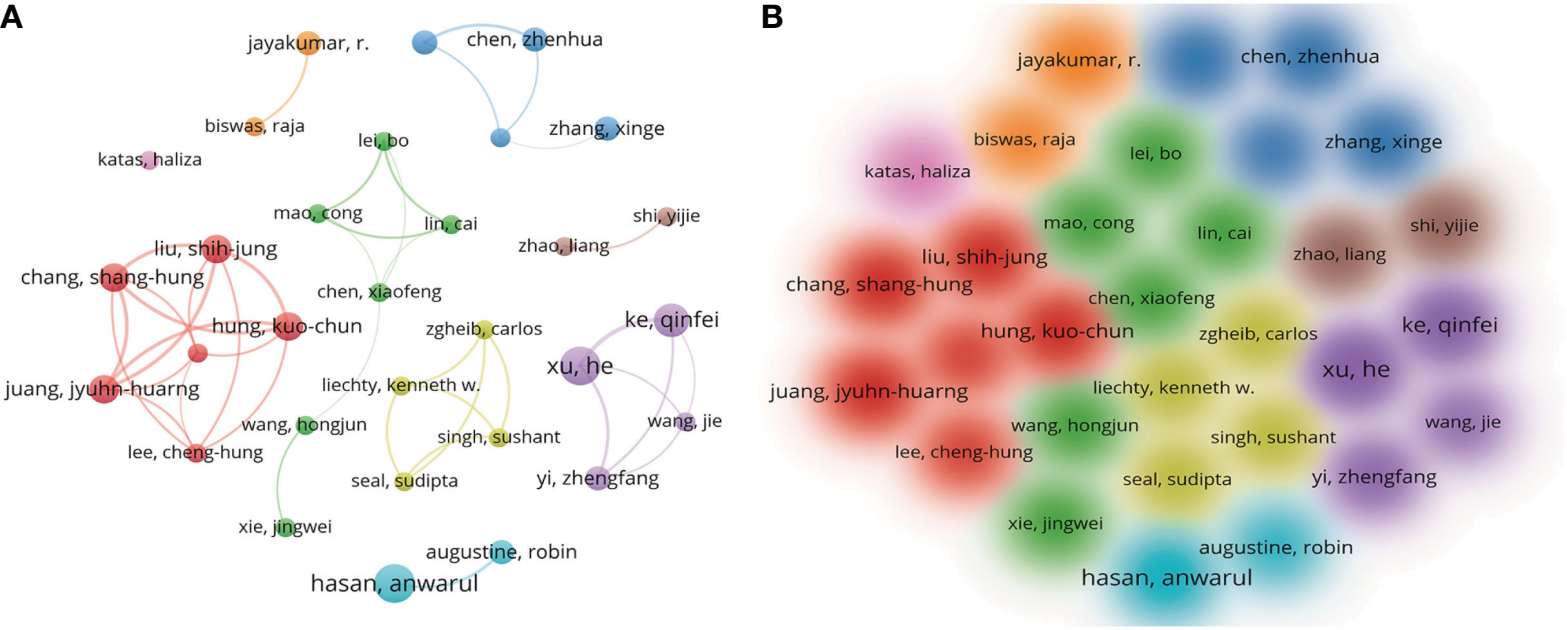
VOSviewer network map of authors **(A)**; Density distribution map of authors **(B)**.

H-index, g-index, and m-index were used to evaluate the academic level and influence of researchers, indicating that Hasan Anwarul made great achievements in nanomaterials and diabetic wound healing-related studies. VOSviewer was used to perform a visual analysis of the co-author (**Figure 6**). The minimum number of articles published by the author was set to 4. The largest clusters of connected authors consisted of six authors, and four of them were in the top 13.

### 3.5 Journals

These articles on nanomaterials and diabetic wound healing were published in 161 journals. **Table 4** lists the top 15 journals with the most articles published, accounting for 38.14% (156/409). The International Journal of Biological Macromolecules, with 250 articles published, was the most productive journal, followed by Materials Science & Engineering C-Materials for Biological Applications currently known as Biomaterials Advances (n = 16, IF = 8.457), Acta Biomaterialia (n = 14, IF = 10.634). Based on the co-citation count, the International Journal of Biological Macromolecules (1217 citations), Acta Biomaterialia (871 citations), Biomaterials (581 citations), and Materials Science & Engineering C-Materials for Biological Applications (560 citations) were the top four journals. The others had <500 citations. Besides, 9 of 15 were found in the first quartile (Q1), and four had an IF impact of >10.

**Table 4:** The top 15journals in the field of nanomaterials and diabetic wound healing.

Journal title	Publications	Total citations	Average citation per paper	Impact factor(2021)	JCR	Country
Int J Biol Macromol	22	1217	55.31	8.0252	Q1	Netherlands
Mat Sci Eng C-Mater	16	560	35	8.4572	Q1	Netherlands
Acta Biomater	14	856	61.14	10.6335	Q1	England
J Mater Chem B	13	371	28.54	7.5711	Q1	England
Acs Appl Mater Inter	12	747	62.25	10.3831	Q1	USA
Biomater Sci-Uk	9	246	27.33	7.5896	Q1	England
J Drug Deliv Sci Tec	9	90	10	5.0619	Q2	France
Acs Biomater Sci Eng	8	219	27.375	5.395	Q2	USA
Chem Eng J	8	156	19.5	16.7438	Q1	Switzerland
Int J Nanomed	8	159	19.875	7.033	Q1/Q2	New Zealand
Rsc Adv	8	166	20.75	4.036	Q2	England
Sci Rep-Uk	8	261	32.625	4.996	Q2	England
Biomaterials	7	581	83	15.304	Q1	Netherlands
Colloid Surface B	7	244	34.85	5.999	Q1/Q2	Netherlands
Int J Pharmaceut	7	314	44.85	6.51	Q1	Netherlands

**Figure 7:**
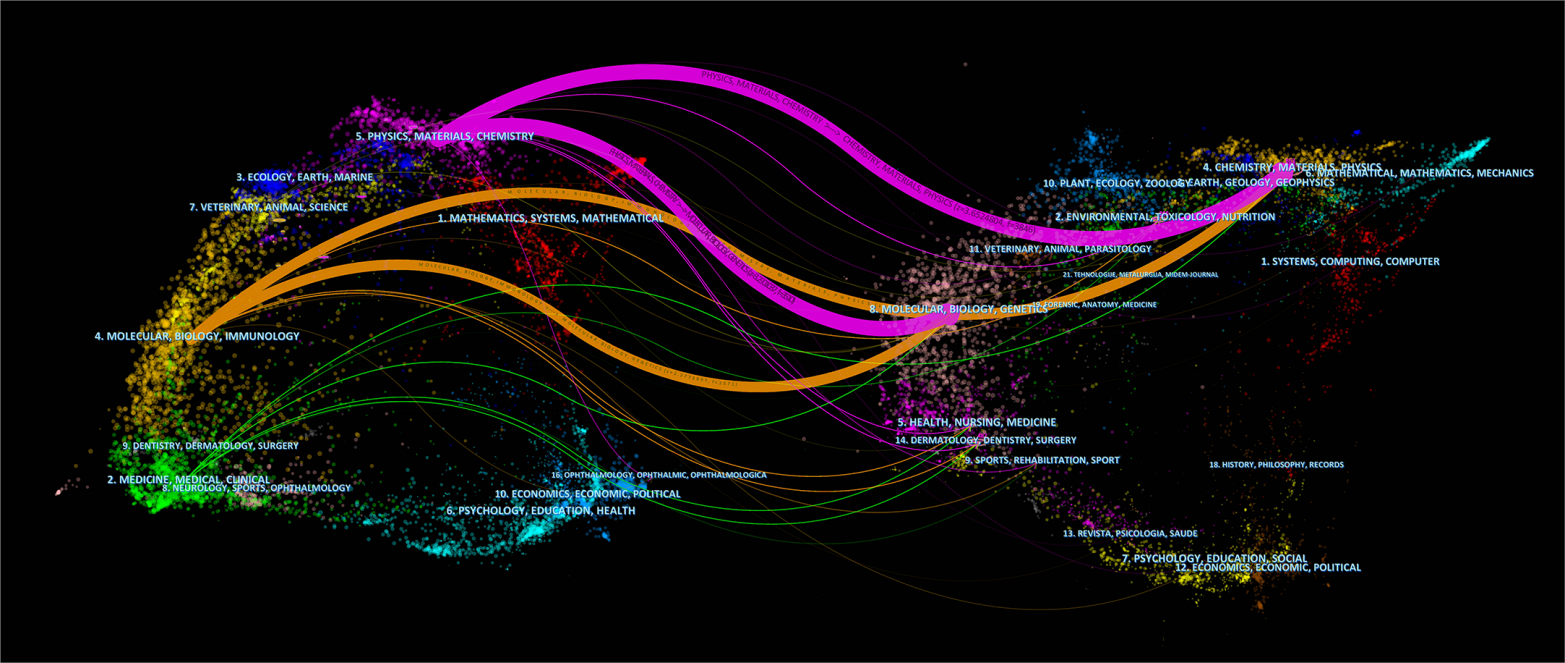
The dual-map overlay of journals contributed to publications regarding nanomaterials for diabetic wound healing from 2011 to 2021.

Citespace was used to show the topic distribution of relationships between journals by using the dual-map overlay. **Figure 7** shows that the citing and cited journals are found on the left and right, respectively, and the colored line path represents the citation relationship, indicating the citation trajectory and knowledge flow. The map showed that molecular/biology/immunology journals generally cited articles published in molecular/biology/genetics and chemistry/materials/physics journals. The two pink routes showed that articles published in physics/materials/chemistry journals often cited molecular/biology/genetics journals and chemistry/materials/physics journals, indicating that articles on nanomaterials for diabetic wound healing mainly focused on journals in molecular, biology, immunology, and physics, materials, and chemistry.

### 3.6 Keywords

**Figure 8:**
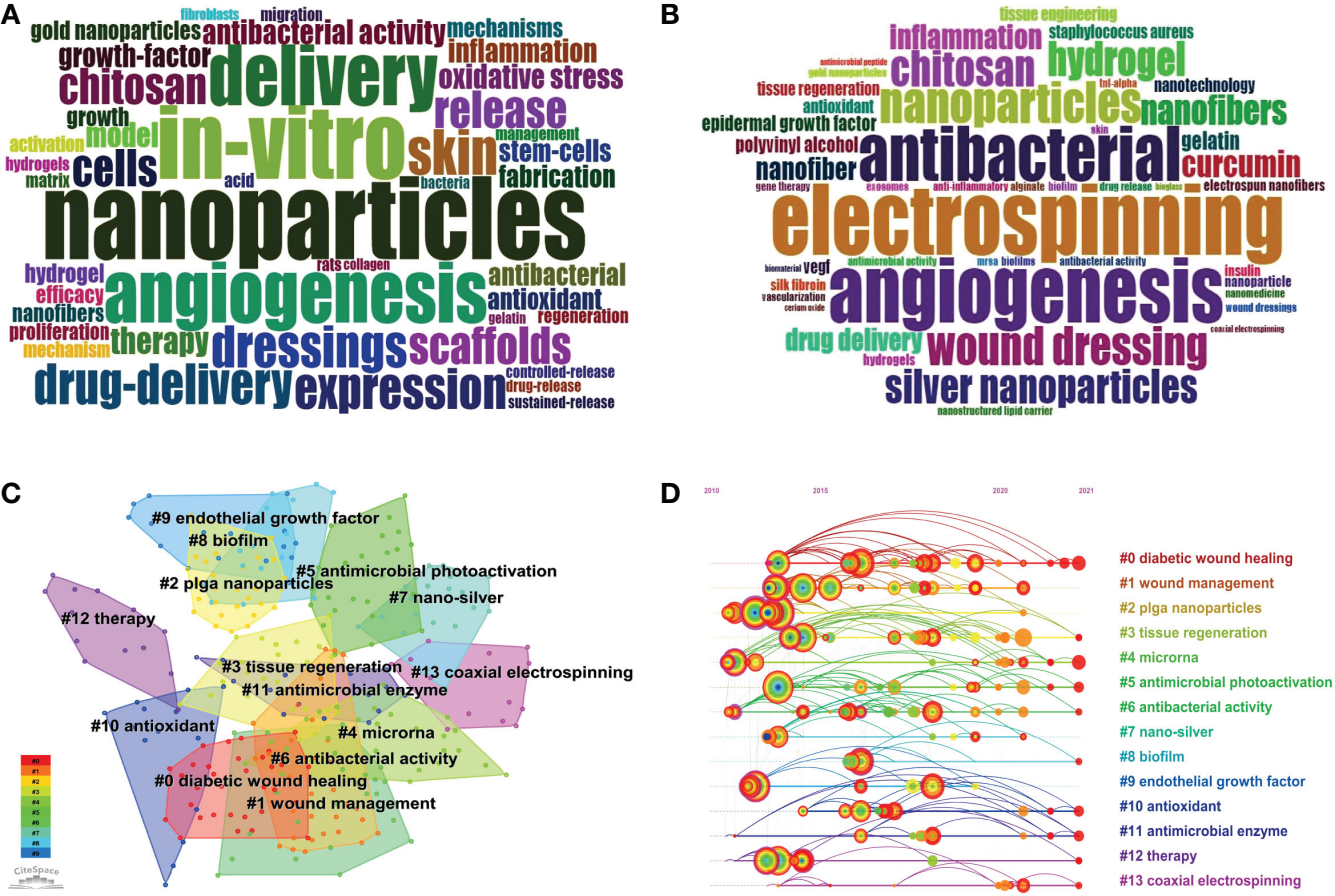
Word cloud of top 50 plus keywords **(A)**; Word cloud of top 50 author’s keywords **(B)**; Map of keyword clusters **(C)**; Timeline view of keyword co-occurrence **(D)**.

The research hotspot in a certain knowledge field can be identified through keyword analysis. Subsequently, 1918 keywords were extracted from 409 selected articles, approximately 4.6 keywords per article. Of these, 172 keywords appeared over 5 times. After removing basic search terms, such as “wounds” and “diabetes,” bibliometrics was used to create a word cloud map for keywords plus and author keywords (**Figures 8A, B**). “Electrospinning,” “angiogenesis,” “antibacterial,” “nanoparticles,” “drug-delivery,” and “wound dressing” were common research hotspots in nanomaterials and diabetic ulcer healing.

In terms of keyword co-occurrence, CiteSpace software was used to perform keyword clusters by using the LLR algorithm. We selected the first 14 cluster labels (#0–#13) for analysis. The 14 clusters focused on 3 aspects: (1) diabetic wound healing process, including clusters #0, #1, #3, #4, #9, and #12; (2) nano-sized nanomaterials, including clusters #2, #7and #13; (3) mechanism of nanomaterials promoting diabetic wound healing, including clusters #5, #6, #8, #10 and #11.

**Figure 9:**
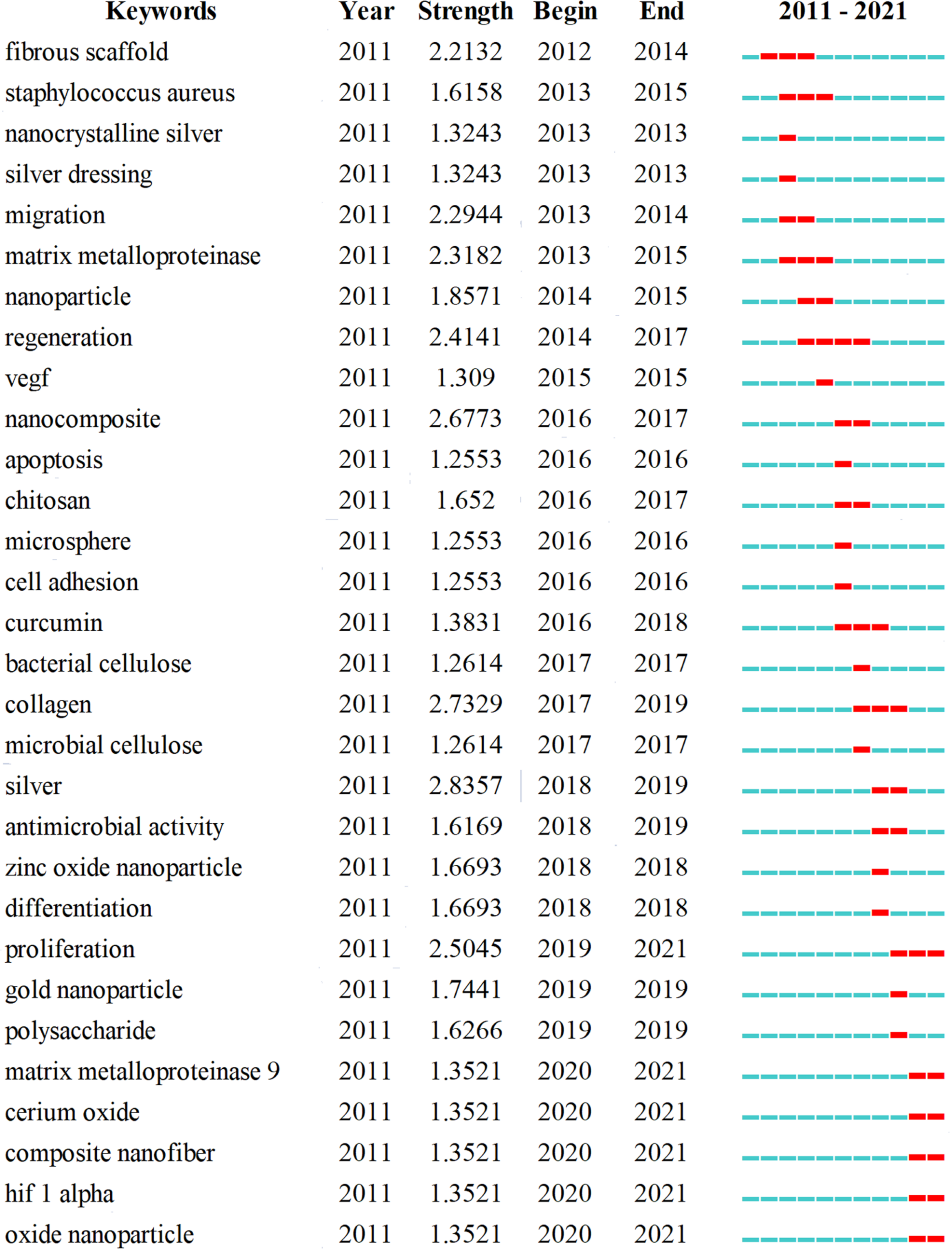
Top 30 keywords with the strongest citation bursts.

The clustering time map can further show the appearance of each cluster. The end and time trends can reflect the importance, degree, and distribution period of a cluster. We found that cluster #9 stopped evolving since 2019. Although cluster #8 appeared the latest, it continued to evolve recently. In addition, clusters #5, #6, #10, and #11 maintained a certain heat recently, indicating that the mechanism of nanomaterials in treating diabetic wounds is the current research trend. Moreover, keyword bursts were used to show research trends in the field. **Figure 9** shows the top 30 most cited keywords. The keyword with the highest burst intensity was “silver” (2.8357), followed by “collagen” (2.7329) and “nanocomposite” (2.6773). The keyword with the longest duration was “regeneration”, followed by “fibrous scaffold”, “*Staphylococcus aureus*”, “matrix metalloproteinase”,”curcumin”, “collagen”, and “proliferation.”

### 3.7 Citation and co-cited reference

Citations are the standard way for authors to indicate the source, ideas, and findings of their research methods. The citation frequency of an article can measure its value to a certain extent. **Table 5** shows the top 10 globally cited articles (23–32). “Engineering Bioactive Self-Healing Antibacterial Exosomes Hydrogel for Promoting Chronic Diabetic Wound Healing and Complete Skin Regeneration” published in Theranostics in 2019 had the largest number of citations (289 times).

**Table 5:** Top 10 global cited articles in nanomaterials and diabetic wound healing.

Title	Journal title	First authors	Year	Total Citations
Engineering Bioactive Self-Healing Antibacterial Exosomes Hydrogel for Promoting Chronic Diabetic Wound Healing and Complete Skin Regeneration (23)	Theranostics	WANG CG	2019	289
Promotion of skin regeneration in diabetic rats by electrospun core-sheath fibers loaded with basic fibroblast growth factor (24)	Biomaterials	YANG Y	2011	256
Skin-inspired antibacterial conductive hydrogels for epidermal sensors and diabetic foot wound dressings (25)	Advanced Functional Materials	ZHAO Y	2019	219
Fibrin-based scaffold incorporating VEGF- and bFGF-loaded nanoparticles stimulates wound healing in diabetic mice (26)	Acta Biomaterialia	LOSI P	2013	217
Tailored design of electrospun composite nanofibers with staged release of multiple angiogenic growth factors for chronic wound healing (27)	Acta Biomaterialia	LAI HJ	2014	201
Novel electrospun chitosan/polyvinyl alcohol/zinc oxide nanofibrous mats with antibacterial and antioxidant properties for diabetic wound healing (28)	International Journal of Biological Macromolecules	AHMED R	2018	192
A cooperative copper metal-organic framework-hydrogel system improves wound healing in diabetes (29)	Advanced Functional Materials	XIAO JS	2017	175
Efficient angiogenesis-based diabetic wound healing/skin reconstruction through bioactive antibacterial adhesive ultraviolet shielding nanodressing with exosome release (30)	ACS Nano	WANG M	2019	169
Combined effect of PLGA and curcumin on wound healing activity (31)	Journal of Controlled Release	CHEREDDY KK	2013	167
Copper metal-organic framework nanoparticles stabilized with folic acid improve wound healing in diabetes (32)	ACS Nano	XIAO JS	2018	163

**Figure 10:**
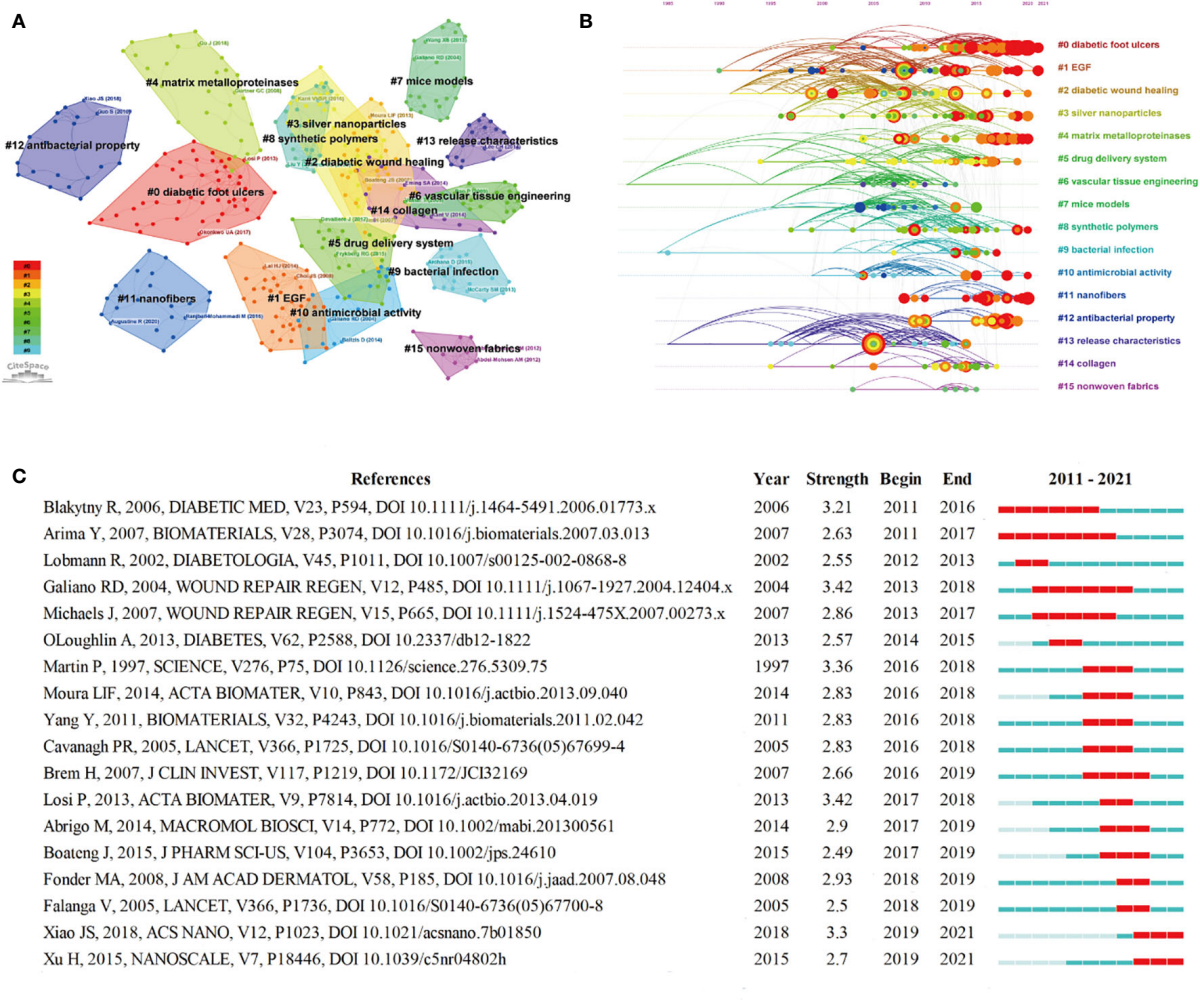
Map of cited references clusters **(A)**; Timeline view of cited references co-occurrence **(B)**; Top 18 references with the strongest citation bursts **(C)**.

Reference co-citations can be used to measure the degree of mutual influence among studies, among which the highly cited studies form the disciplinary basis of this field. **Table 6** lists the top 10 co-cited references (26, 33–41). The most co-cited reference was “Wound healing and its impairment in the diabetic foot” by Falanga et al. in 200530. All references were clustered into the major 16 clusters, including “#0 diabetic foot ulcer”, “#1 EGF,” “#2 diabetic wound healing,” and “#3 silver nanoparticle,” and so on (**Figure 10A**). **Figure 10B** shows that “#11 nanofibers” and “#13 antibacterial properties” continuously evolved.

**Table 6:** Top 10 co-cited references in nanomaterials and diabetic wound healing.

Title	Journal title	First authors	Year	Total Citations
Wound healing and its impairment in the diabetic foot (33)	Lancet	FALANGA V	2005	65
Cellular and molecular basis of wound healing in diabetes (34)	Journal of Clinical Investigation	BREM H	2007	47
Factors affecting wound healing (35)	Journal of Dental Research	GUO S	2010	38
In vivo wound healing of diabetic ulcers using electrospun nanofibers immobilized with human epidermal growth factor (EGF) (36)	Biomaterials	CHOI JS	2008	32
Recent advances on the development of wound dressings for diabetic foot ulcer treatment--a review (37)	Acta Biomaterialia	MOURA LIF	2013	29
Wound repair and regeneration (38)	Nature	GURTNER GC	2008	28
Wound healing dressings and drug delivery systems: a review (39)	Journal of Pharmaceutical Sciences	BOATENG JS	2008	26
Fibrin-based scaffold incorporating VEGF- and bFGF-loaded nanoparticles stimulates wound healing in diabetic mice (26)	Acta Biomaterialia	LOSI P	2013	26
Diabetes and wound angiogenesis (40)	International Journal of Molecular Sciences	OKONKWO UA	2017	24
Cutaneous wound healing (41)	New England Journal of Medicine	SINGER AJ	1999	24

**Figure 10C** shows the top 18 references with the strongest citation bursts. “Fibrin-based scaffold incorporating VEGF- and bFGF-loaded nanoparticles stimulate wound healing in diabetic mice” by Losi et al. in 2013, and “Quantitative and reproducible murine model of excisional wound healing” by Galiano et al. in 2004 had the strongest burst (3.42) (26, 42). Additionally, the article “Effect of wettability and surface functional groups on protein adsorption and cell adhesion using well-defined mixed self-assembled monolayers” by Arima et al. had the longest burst period from 2011 to 2017 (43).

## 4 Discussion

Non-healing diabetic wounds, resulting in high mortality rates and disability in diabetic patients, have become a global public health problem. Over the past years, many studies on the applications of nanomaterials in diabetic wound healing have been conducted to confirm that nanomaterials are effective pathways in treatment. Bibliometrics help researchers make comprehensive cognition in the field. For the first time, different bibliometrics software were combined to sort out the current characteristics and predict the development trends of nanomaterials and diabetic wound healing.

### 4.1 General information

The number of publications reflect the scientific activities in a field. Based on our study, 2508 authors from 699 institutions in 51 countries or regions have contributed 409 publications to the field of nanomaterials and diabetic wound healing from 2011 to 2021. Moreover, the global volume markedly increased. The number of annual output is almost twice as high as that in the previous year, indicating a promising prospect in the research regarding the application of nanomaterials in diabetic wound healing.

China is the most productive country, with 167 articles in terms of country publication volume. Compared with China, publications from other countries significantly decreased. China and the USA have the closest collaboration, indicating that China is the core country for international research on nanomaterials and diabetic wound healing. In addition, India has the highest MCP ratio in the top three productive countries. Moreover, India plays an important role in international cooperation. As the two most populous countries in the world, China and India have the highest number of diabetic patients. Thus, scholars are driven to explore the application of nanomaterials in diabetic non-healing wounds.

Furthermore, 699 institutions contributed articles to the application of nanomaterials in diabetic wound healing. The most prolific institution was Shanghai Jiao Tong University from China which published nine papers in 2021. **Figure 6** shows that institutions in close geographical proximity usually collaborated actively. For example, no cooperation was found between Shanghai Jiao Tong University and Univ Tehran Med Sci, although they were ranked at the top. Regarding the author’s contribution, Chinese scholars still played the lead role in publishing articles in the field. **Table 3** shows that the most prolific authors were Hasan Anwarul. The latest article by Hasan Anwarul indicated that EGF-loaded PHBV-Gel MA hybrid patches could accelerate wound healing in diabetic mice by promoting the migration and proliferation of multiple cell types and angiogenesis (44). Similar to the status of the institutions’ links, many cooperating groups exist among several scholars. To develop this field, institutions and researchers should focus on similar research topics to establish closer cooperation.

### 4.2 Research trends and hotspots

Keyword evolution and cited references map the dynamic changes in research hotspots. Therefore, we conducted multiple visualization knowledge mapping, including cluster analysis, timeline view, and citation bursts of the references and keywords. Analysis of keywords and co-cited references showed that nanomaterials, such as oxide nanoparticles with antibacterial and antioxidant qualities and promoting angiogenesis, are encouraging trends.

Nanotechnology is an emerging field in treating diabetic complications. Nano-sized materials, such as nanoparticles, liposomes, nanofibers, and hydrogel, have an advantage in delivering drugs, providing sustained release, prolonging action time, and reducing side effects (45, 46). Nanomaterials are ideal carriers for enhancing drug delivery efficiency and cell regeneration. Researchers showed great interest in these topics from 2011 to 2015. **Figure 9** shows that “fibrous scaffold,” “nanocrystalline silver,” and “nanoparticle” appeared in the earliest burst keywords, as well as “migration” and “regeneration,” which are important terms in progress in wound healing. **Table 6** lists the top ten co-cited references. Most were reviews and aimed to outline the physiological and pathological processes of wound healing and summarize the negative factors that delay wound healing. Two original studies in the top ten co-cited references developed suitable carriers to deliver drugs or cytokines, such as EGF, VEGF, and bFGF (26, 36).

In addition, nanomaterials with antibacterial, anti-inflammatory, and antioxidant properties are another main reason for their application in improving diabetic wound healing. **Figures 8C, D**, **10A, B** show that antibacterial or antimicrobial is the most important cluster that continuously evolves in diabetic wound healing. Besides, the ongoing burst keywords included “oxide nanoparticle,” “hif 1 alpha,” and “cerium oxide” which reflected the latest research trends. HIFP-1α is an important factor in diabetic wound healing, and the degradation of polyubiquitinated HIF-1α diminishes wound healing efficacy. Therefore, it is an effective treatment to stabilize the HIF-1α protein. Yang et al. designed an intelligent NIR-triggered NO nanogenerator to increase HIF-1α expression to enhance angiogenesis (47). Leung et al. also Introduced a small-molecule HIF-1α stabilizer to accelerate diabetic wound healing by enhancing angiogenesis (48). Metals and their ions were beneficial to wound care. With the development of nanotechnology, metal oxide nanoparticles have attracted the interest of researchers. Cerium oxide nanoparticles were widely used in diabetic non-healing wounds because of their antioxidant properties. Augustine et al. designed a type of cerium oxide nanoparticle-loaded gelatin methacryloyl hydrogel to accelerate wound healing in diabetic mice (49). Dewberry et al. and Stager et al. also used cerium oxide nanoparticles as raw material in preparing a nanocarrier to promote diabetic wound healing (50, 51). Some metal oxide nanoparticles were utilized because of their antibacterial properties. Wang et al. developed a nanocomposite scaffold composed of iron oxide nanoparticles and showed antioxidant and substantial antibacterial properties in an *in vitro* study (52). Nanocomposites, including zinc oxide and magnesium oxide, seem to promote wound healing through their antibacterial activity (53, 54). Thus, developing materials with powerful antioxidant and antibacterial activities is challenging for researchers.

## 5 Conclusion

To the best of our knowledge, this is the first bibliometric analysis that investigated the application of nanomaterials in diabetic non-healing wounds. A comprehensive survey and analysis of countries, authors, keywords, journals, and literature was conducted. Bibliometric networks through authors, institutions, co-citation, and co-occurrence analyses were constructed using various scientific tools to reveal the bibliometric characteristics of the field. Moreover, the degree of international cooperation should be enhanced to develop this field. Nanomaterials with antibacterial and antioxidant qualities and promoting angiogenesis are the current research topics.

## Data availability statement

The original contributions presented in the study are included in the article/supplementary material. Further inquiries can be directed to the corresponding author.

## Author contributions

Conception and design: ZW, JZ. Data collection and interpretation: JZ, TC, HL. Data analyses: YZ, XN. Manuscript draft and critical review: all authors. Final approval of the study content and manuscript and accountability for data integrity: all authors. All authors contributed to the article and approved the submitted version.
